# Psychoanalysis and Interdisciplinarity With Non-analytic Psychotherapeutic Approaches Through the Lens of Dialectics

**DOI:** 10.3389/fpsyg.2021.697506

**Published:** 2021-07-08

**Authors:** Yael Peri Herzovich, Aner Govrin

**Affiliations:** Interdisciplinary Studies Unite, The Program for Hermeneutics and Cultural Studies, Bar-Ilan University, Ramat Gan, Israel

**Keywords:** psychoanalysis, interdisciplinarity, dialectics, Hegel, philosophy of psychoanalysis

## Abstract

Psychoanalysis, in its purist mainstream sense, tends to be considered as an isolationist discipline that steers clear of interdisciplinary connections with other psychotherapies. Its drive for purity does not open up to influences that cast as alien and a threat to its core principles. We refer to Hegelian dialectics in an attempt to offer an alternative approach to interdisciplinarity in clinical psychoanalysis. Psychoanalysis entertains a complex dialectical relationship with the major theories it opposes. In this dynamic, psychoanalysis begins by negating the non-psychoanalytic theory as a part of self-negation (Hegel calls this phase self-alienation). But in its own process of growth, it negates this negation and reabsorbs the alienated self part. Reabsorbing the negated component, psychoanalysis does not revert to its original identity but becomes sublated into a different, more complex idea. In this epistemological process, psychoanalysis deals with its own practical and theoretical anomalies and lacunas. The paper illustrates this process using three central developments in the history of psychoanalysis: empathy in self psychology (connection with Rogers' humanist psychology), short-term dynamic psychotherapy (connection with short, intensive therapies), and mentalization-based psychotherapy (connection with cognitive-behavioral therapies). In all of these cases, psychoanalysis integrates components it previously opposed and changes these components to their own, specific characteristics. We address the epistemological shifts in the scientific status of psychoanalysis and show their connection to dialectics. Finally, we conclude that dialectical development is what allows psychoanalysis to remain relevant and up to date, to be open to interdisciplinary influences without its identity and tradition coming under threat.

## Psychoanalysis and the Extra-Analytic Other

In recent years there were several attempts to characterize the evolution of psychoanalytic thought. Makari (2000) posits that contemporary psychoanalysis, with its numerous models of mind and psychopathology, includes both a far-reaching and vital Kuhnian proto-science, as well as a historically deep practice of meanings and values. Makari believes that change in psychoanalysis is not uniform. Rather, some progress in psychoanalysis reflects the kinds of shifts Kuhn ascribed to a proto-science and change that propose a new net of meanings, an ethic, a way to live in the world. Govrin (2004) argues that shifts in epistemological assumptions have always accompanied significant ideological nodes in the history of psychoanalysis. In other words, the psychoanalytic therapeutic concepts have changed because the cultural and philosophical context of the world view has changed, mainly from positivism to post-modernism.

In this paper, we would like to describe the evolution of psychoanalysis by using Hegel's dialectic process stages. But let us first clarify what we mean when using the broad term “psychoanalysis” and what we do not mean.

The field of psychoanalysis has gone through many theoretical evolutions since Freud's time, from an emphasis on the drives to ego psychology, object relations theory, self psychology, and is currently preoccupied with post-modern perspectives and those focusing on relationality and intersubjectivity. Therefore, psychoanalysis is not (anymore) a “monolith” but includes within it a multitude of different positions. These include ones that seek to integrate psychoanalysis and other non-analytic approaches (such as neuropsychoanalysis, infant research, and integration with CBT and other orientations). It is important to note that in this paper, we use “psychoanalysis” in its purist mainstream sense by which the nucleus of the identity of psychoanalysis lies in the “understanding” of unconscious dynamics (Migone, 2011) and uncovering and understanding the (often unconscious) conflicts and early developmental experiences associated with the client's symptoms (Boswell et al., 2010). We are aware that many other forms of psychoanalysis are oriented toward integration and mutual influence with non-analytic theories (they are usually heavily criticized by the “purists” [see for example, Blass and Carmeli (2007) criticism of neuropsychoanalysis, and Green (2000) criticism against infant research]. We are also aware that in many contemporary psychoanalytic approaches, there is not that a rigid opposition between psychoanalysis and non-psychoanalysis, being themselves not considered psychoanalytic by classical or more conservative psychoanalysts (see for examples the intersubjective or the relational tracks, etc.). However, we believe that mainstream psychoanalysis is still a dominant force, especially today when psychoanalysis needs to show its relevancy in a world that offers many other effective therapies. Furthermore, psychoanalysis still often defines itself by reference to what it is not, which means other psychotherapeutic schools.

This paper will therefore concentrate on a specific phenomenon or trend within mainstream psychoanalysis that reflects itself within many psychodynamics clinicians (Govrin, 2015)- analyst's indifference to non-analytic approaches. This is reflected by: (a) Psychoanalysis flagship journals tend to be “purists” and to overlook important developments in other fields. (b) training in many psychoanalytic institutes is essentially non-integrative. Cherry et al. (2020) conducted interviews with 69 graduates from the Columbia University Center for Psychoanalytic Training and Research since 2003. It seems that the non-analytic world had little if any place in their psychoanalytic education. Furthermore, one of the significant aims of training of the twenty first century is to show the advantage of psychoanalysis over other approaches. Fritsch and Winer (2020), in their propose “a Model of Psychoanalytic Education for the Twenty-First Century,” write:

“Training the fertile fields of the 1950's had become the desert of the 2000's. A popular explanation was that we had lost cultural currency. We had been replaced both by new therapeutic approaches that promised greater benefits, faster and at a fraction of the price—CBT, DBT, Prozac—and by other sorts of mental and physical approaches: yoga, mindfulness training, EMDR.”(p. 175). (c) case studies are almost exclusively pure psychoanalytic and rarely integrate other ideas from non-analytic theories.

The indifference to non-analytic theories is reflected by a dismissive approach, sometimes by criticism (Westen et al., 2004; Shedler, 2015) and most often by overlooking it. Govrin (2015) has explained the relative indifference of analysts to other theories by showing that analysts use narratives that are coherent all-encompassing, and useful even when therapeutic failures occur. Alternative theories simply do not fit the coherence of the narrative and therefore are of no use. A Foucauldian perspective also sheds light on psychoanalysis' wholeness: “Psychoanalysis is the term by which we designate one of the disciplines among the psychological and social sciences, a discipline that includes a taken-for-granted understanding of the human subject and a therapeutic technology for its management. The assemblage that comprises psychoanalysis as a discipline entails a particular discourse on human existence, a life-and identity-defining master narrative which articulates a specific form of the subject that is asserted to be natural, essential, ahistorical, and universal” (Milchman and Rosenberg, 2011, p.6).

Rejection to empirical findings and to new paradigms are widespread in science. Many post-positivist philosophers of science described scientist's resistance to change even in the face of contradicting findings. Cohen (1985), for example, writes:

“The desire to be an active part of a revolutionary movement is often in conflict with the natural reluctance of any scientist to jettison the set of accepted ideas on which he has made his way in the profession. New and revolutionary systems of science tend to be resisted rather than welcomed with open arms because every successful scientist has a vested intellectual, social, and even financial interest in maintaining the status quo” (p. 35).

In *The Origins of Modern Science*, Butterfield (1997) argued that “the most difficult mental act of all is to rearrange a familiar bundle of data, to look at it differently and escape from prevailing doctrine” (p. 106). He also writes that “of all forms of mental activity, the most difficult to induce even in the minds of the young, who may be presumed not to have lost their flexibility, is the art of handling the same bundle of data as before but placing them in a new system of relations with one another by giving them a different framework” (p. 13).

While rejecting evidence and resistance to change are features characterizing science at the descriptive level of discourse, science must be open to new evidence and change at the prescriptive level.

Psychoanalysis (in the sense we use it here) tends to be considered an isolationist discipline that makes few interdisciplinary connections with other psychotherapies. There is a common belief that psychoanalysts interact almost exclusively with each other (Malcolm, 1982). By doing so, they deprive themselves of exposure to competing viewpoints and alternative perspectives that might enrich the psychoanalytic model (Bornstein, 2001). Training programs, major journals in the field, conferences, and the general psychoanalytic discourse invest little effort in non-analytic clinical theories and the many possibilities the introduction of some of their ideas might hold. The approach surrounds itself with a faithful community of professionals who identify with it and often define themselves by contrast with other therapeutic streams (Safran and Messer, 1997). Many scholars who reflect on this phenomenon usually think that a therapist from another therapeutic persuasion is regarded as belonging to a foreign culture (Wachtel, 2010) and that the other functions to define and maintain self-definition and the values of the approach (Sampson, 1993). The distinction between us vs. them helps to consolidate those who follow the method and give them political power (Sorenson, 2000).

Loyalists' main worry is that psychoanalysis, once exposed to another, alien direction, will not manage its own tradition's mainstay, like bringing the unconscious to consciousness. As a result, the profession, it is feared, may cave in before superficial, intense, and fast therapies [e.g., Blass (2010) and Berman (2010), response]. Psychoanalysis's motivation for maintaining the tradition's mainstay in therapeutic theory and practice consists of many reasons (some mentioned above). First, psychoanalysis is a theory and technique for treating psychological disorders; it deals with the relief of mental suffering. Its followers believe its ideas about what can count as an effective therapy (Wachtel, 2018). Second, it also involves economic competition over public resources, recognition, and prestige (Miltone, 2001; Shahar, 2011). Also, a fear of questioning identity and the wish to maintain a solid and robust identity is another reason why boundaries between schools are required (Peri Herzovich and Govrin, 2021).

Any object that threatens us must be an object which wse already recognize as relevant to us, as being in some relation to us. Not everything outside us is experienced as a threat: An alternative psychotherapeutic approach may be perceived as a threat by psychoanalysis, where a new mathematical model won't. Kristeva (1991/1988) argued that what we consider foreign – which manifests as hatred of the other – includes a hidden aspect of our own identity. The difficulty we have in accepting the other, she believes, is the outcome of our inability to acknowledge our own subjective otherness. As a result, we experience those unlike us as a threat and need to keep them out. Kristeva refers to the Freudian unconscious in her description of the hatred of the stranger as a manifestation of the unconscious projection and rejection of uncontrollable drives or unprocessed parts. In his writing about the uncanny, Freud puts this as follows: “[…] for this uncanny is in reality nothing new or alien, but something which is familiar and old-established in the mind and which has become alienated from it only through the process of repression” (Freud, 1919, p. 241). And so what is alien and threatening are materials that have undergone repression and exclusion processes to be removed from ourselves. That is to say; there is both a process of repression and a process of externalization of self components, both of which are done unconsciously. The common result is that what I experience as a stranger and as an other is not only his or her own foreignness but actually a foreignness of myself. This is why in this paper, we argue that the alienness of non-psychoanalytic approaches, in addition to forming an external threat, also represents the threat from within.

In this paper, we would like to add another aspect to the relations between mainstream psychoanalysis and non-analytic theories to show the dialectic nature of their relations. We refer to Hegel's epistemology (epistemology as the study of knowledge) to show that psychoanalysis evolves dialectically by integrating or assimilating external influences like other bodies of knowledge that also represent reabsorbing alienated self-parts.

Hegel was one of the first historical epistemologists. His dialectics is a general developmental theory of the subject, but it is particularly relevant to the development of knowledge. If we look at psychoanalysis's history through the lens of dialectics, we find that psychoanalysis' attitude to other therapeutic methods is more complicated than meets the eye. We perceive the quest for purity in psychoanalysis as a form of self-negation. Still, we believe it is only part of an entire dialectical process by which psychoanalysis does uniquely incorporate external components. While the dialectic process of affirmation of identity and becoming is unconscious, what is conscious is the consideration of other approaches as alien and extraneous to the psychoanalytic field.

Although we cannot give a complete representation of Hegel's dialectic in a psychological journal, it is possible to capture *some* of the dialectics' essential features that will shed new light on the intricate relations between psychoanalysis and other psychotherapies. According to Mills (2012), what is central to Hegel's overall philosophy is the notion of process, a thesis that has direct implications for the development of psychoanalysis. But “One does not have to espouse Hegel's entire philosophical system, which is neither necessary nor desirable, in order to appreciate the dialectic and its application to psychoanalysis and contemporary modes of thought”(p. 188).

Dialectic development in psychoanalysis occurs when an initial component of it undergoes *negation*, is rejected, and projected onto the therapeutic other (Hegel calls this phase self-alienation). Negation can take the form of criticism or total disregard, and it serves to preserve the clear identity of the theory. Over time, however, when the approach fails to offer a sufficient response to clinical challenges, *a negation of the negation*, the next step in the dialectical process, takes place. The previously cast-off, negated part is restored to the theory to deal with the perceived lack. However, as it performs this negation, it dialectically produces a synthesis with the negated component on a higher level, namely by including it, theoretically and/or practically, in a new guise. When this negated element is incorporated in the mainstream, by negating its negation, it does not retain its original identity. This is where Hegel's notion of *Aufhebung* or sublation comes in: the newly integrated element produces a more complex and different idea. While sublation negates and rejects the negated component, it also preserves that component's essence, thereby raising psychoanalysis to a higher level. In this manner, we can describe how psychoanalysis develops in a dialectic process that tends to perfection, a stage which Hegel called Absolute Knowledge.

Hegel's idealism seeks to offer a total and absolute account of the development of the subject and of knowledge that was appropriate to his times. In our post-modern reality, such total idealist theories have become controversial. We suggest focusing on Hegel's description of the dialectical dynamic through which both subject and knowledge emerge, taking an epistemic perspective. This, we believe, yields a new way of looking at the history of psychoanalytic thinking. Considering this history as dialectical, we perceive it as interdisciplinary in essence. Such an approach resolves the tension in psychoanalysis' attitude to other psychotherapeutic disciplines because it shows how it needs them to constitute its own distinct and separate identity.

It is important to note that every human endeavor might be represented as interdisciplinary in essence. However, among different disciplines, psychoanalysis's inclination toward interdisciplinary is remarkable since it touches on so many different aspects: science, hermeneutics, biology, development, brain research, philosophy, art, and humanities. Still, the interdisciplinary relationship between psychoanalysis and other non-analytic psychotherapeutic approaches has not been sufficiently explored.

In the first part of this paper, we present Hegelian dialectics to explain how scientific knowledge develops. We shall limit ourselves to some of its central and important concepts. In the second part, we put these concepts to use in describing three major developments in psychoanalysis: the introduction and incorporation of self psychology, including the notion of empathy (by way of linking with Rogers' humanist psychology); short-term dynamic psychotherapy (by way of linking with intensive therapies), and mentalization-based therapy (by way of linking with cognitive-behavioral psychology). In the third part, we refer to the scientific status of psychoanalysis and shifts in epistemological positioning. In the concluding section, we will discuss the importance of dialectic processes in maintaining psychoanalysis' vitality.

It is important to note that Hegel's dialectical process can describe the evolution of all psychotherapies such as CBT, Gestalt, Family System Theory, and Emotion-Focused Therapy. Indeed, Hegel takes dialectic to be a general theory of development. Hegel (1892b) says that “wherever there is movement, wherever there is life, wherever anything is carried into effect in the actual world, there Dialectic is at work.” (p. 148). Govrin (2016) described how CBT endorsed mindfulness, a spiritual Zen practice, and incorporated it within its rational scientific worldview by a process called “integration through conversion.”

As in psychoanalysis, a similar diversity could be found in other psychotherapy movements, too (Castiglioni and Corradini, 2011). For example, there are two strands partially opposed to each other within the systemic movement: (a) the “Philadelphia School,” which since the 1960's has tried to combine the systemic model with psychoanalytic concepts; (b) the “system purists” who reject all contaminations with “intrapsychic” models, in particular psychoanalysis, to focus -in an entirely relational perspective- on the analysis of pathogenic communicative models (Cf. Gurman and Kniskern, 1981–1991s). However, we believe psychoanalysis is perhaps the most interesting case to demonstrate its evolution by the dialectic process because no other psychotherapeutic school was characterized by so much negation, dismissal, and resistance to change, whether through bitter controversies between new and old psychoanalytic schools (See the Freud- Klein controversies, Steiner, 1991) or through dismissing non-analytic theories. Nearly all non-analytic psychotherapies such as client-centered therapy, family system therapy, Gestalt, and CBT evolved through negation of psychoanalysis principles, and it can be demonstrated that through a dialectic process how the negated elements have been incorporated into these systems later in a new guise, but this deserves a separate paper.

## Hegel's Method

Though Hegel's thought is dense and detailed, it is possible to describe the dialectics' main thesis quite concisely. According to Thagard (1982), Hegel elaborated his dialectics concerning consciousness in his *The Phenomenology of Spirit* (Hegel, 2018/1807); in relation to history in his *Philosophy of History* (Hegel, 1956/1837), and more specifically to the history of philosophy in *Lectures on the History of Philosophy* (Hegel, 1892a), and to the logical categories in *Science of Logic* (Hegel, 1969/1812). In the first of these, Hegel elaborates how human consciousness emerges from containing only the most primitive knowledge to having the capacity to attain absolute knowledge. His logical categories describe how a “notion” evolves from the most primitive category – Being – to the overarching category of the Absolute Idea. Every process, Hegel argues, has the same structure. Thus, about consciousness, he writes: “the development of this object, like the development of all-natural and spiritual life, rests solely on the nature of the pure essentialities which constitute the content of logic” (Hegel, 1969/1812, p. 28). Still, one can form a particularly clear understanding of how Hegel's dialectics construes the growth of scientific knowledge from the dialectics of the stages of consciousness, as they appear in the *Phenomenology of Spirit* (Hegel, 2018/1807), compared, for instance, to his pure dialectics of logic [in *Science of Logic* (Hegel, 1969/1812)]: In the former, he foregrounds the human subject's development of knowledge. Our discussion, therefore, is especially relevant to the dialectics of consciousness described in the former text.

Below we will describe the dialectical process of the formation of the subject that also obtains for the process whereby knowledge is consolidated. That will be described after it as the development of a subject-like system.

### Development of the Dialectics of Self-Consciousness

*The Phenomenology of the Spirit* (Hegel, 2018/1807) describes the emergence of the subject, from a *consciousness* whose content is another object to *self-consciousness* – now the object of consciousness is its self. This process, for Hegel, requires another subject. It is in the encounter with the other and through mutual recognition that the two sides constitute themselves as self-conscious subjects.

Hegel begins by distinguishing between subject and object. While the object is a primary, simple given which operates according to the principle of self-identity (A=A), the subject is not aprioriy given and is never identical to itself. The subject constructs its identity and knowledge through a dialectical relationship between difference and identity. This happens in a three-stage, iterative, cyclic process: *identity, the negation of identity* and therefore difference, the *negation of difference* and therefore identity, and hence renewed identity. When the identity between two things is negated, difference, or opposition, between them emerges, and when this is followed by the negation of difference as opposition, identity is re-established. But this third stage, the negation of the negation, does not take us back to where we began. It produces a new, more complex tier of identity. This new level of identity is of a higher order, and it goes by the name dialectical identity. Dialectical identity simultaneously retains the difference between the two terms it includes but also cancels it, allowing their identity (Levkovich, 2011).

A particular content's primary form is negated and canceled in every dialectical process while its fundamental meaning is maintained on a higher level of formalization and expression. This content, preserved as an element in the new condition, comes about through what Hegel called *sublation* [the German word *Aufhebung* literally refers to negation or cancellation and elevation and a movement upward and ahead (Yovel, 2001)]. Each new stage sublates the one before it and replaces it. So sublation is a type of dialectic development including three moments: The negation or cancellation of a given form, retention of the fundamental content, and raising this content to a higher level of expression. Certain components are rejected in this process as essential components are accepted and preserved. Sublation retains and preserves on the one hand while also criticizing earlier forms of thinking and discarding them. It is always a qualitative renewal process, which raises the subject to a higher ontological and epistemological level. This is a dialectical, not a simple linear, analytical mode of development (Yovel, 1975).

Having described dialectical identity and the process of dialectical development, the next question to be addressed is the position of the other. In Hegel's view, the subject's identity emerges through self-alienation, whereby it becomes other than itself (negation). This implies that what is perceived as other always includes a negated part of the primary identity. It is by returning to the self from this otherness (negation of the negation) – in which the self has recognized the other and hence his own internal otherness – that consciousness can come to recognize itself, to evolve into self-consciousness attaining its own realization.

It must be stressed that the other is an actual other and not just the internal otherness of consciousness. On the one hand, it is not the self; it is, on the other, a moment of self-consciousness. Consciousness needs another consciousness to know itself. This can be explained as follows:

Subjective identity, as said, must be considered as an act of self-identification performed by reference to the other, something which is achieved at the end of a process. It is not identical to itself from the start and only approaches itself through repeated negations of its opposites, through the negation of the negation. Again, dialectical development takes off with the act of negation of a part of itself; The subject casts off this part and identifies it in the other who is experienced as foreign to it. When the dialectical process unfolds properly the subject returns to itself through the negation of the negation.

The components of itself which it formerly negated are now identified as so-called *moments* of the self. For the subject to recognize these multiple moments as his own, and at the same time have a unifying pattern of himself, he needs the mediation of the other. This is because the realization of every being in nature is conditional on the existence of the two moments that constitute its full essence: a moment of plurality and a moment of unity. Human consciousness holds both these moments. It contains the essence of Being, yet it also is Being itself and in need of another consciousness that can have its own moments. Unable to validate itself, human consciousness turns to another who will provide its full realization, namely the moment of its plurality and the moment of its unity (Shalgi, 2009).

Self-consciousness, therefore, is possible only where it is reflected in another self-consciousness; the latter serves as the means whereby consciousness knows itself, or, in Hegel's own words: “Self-consciousness is in and for itself, when, and by the fact that it is in and for itself for another self-consciousness; that is, it is only as something recognized” (Hegel, 2018/1807, p. 76, s.178).

### The Dialectical Development of the Subject-Like Structure

As said, Hegel's dialectics describes not only the emergence of the human subject, but also that of subject-like structures. Dialectic logic traces the dynamic structure of mental structures in so far as they are subject-like structures, including the development of knowledge and science (Cohen and Wartofsky, 1984). The subject-like structure evolves through the other – in this case: other bodies of knowledge. Dialectical development is an iterative process that continues until the absolute realization of the subject or the body of knowledge (which constitutes self-consciousness), a condition Hegel calls the Spirit or Absolute Knowledge.

No subject-like structure features one simple and primary identity: its realization must be understood as an act of self-identification through otherness and the other. Whether we are dealing with a human entity or an entity of knowledge, both take the shape of a subject (having self-consciousness), which is never what it is right from the start and rather proceeds toward itself through its opposites: the plural and the other. A knowledge entity, therefore, comes about similarly as a subject, through negation.

Negations do not take the process back to where it began: each act of negation institutes a different state of affairs (and consciousness). Earlier stages are not erased by negation: they are retained in the very texture of the next stage as a type of memory, expressing sublation. At each stage, the collapse of one position advances the process toward another position serving as a specific, even if temporary, response to the specific fault which came to light at the earlier stage and caused its collapse (such faults, in science, are called anomalies, lacunae or unresolved problems). In this process, the entity of knowledge (or the subject-like structure) assumes various forms and contents that are retrospectively considered as expressions of its self. Thus, this is permanently becoming and does not exist in actuality, except for eventually, at the end of the process (if there is such a thing) when it is realized in the complete process and its result (Yovel, 2001).

So for Hegel, a knowledge entity comes into being in the same manner as self-consciousness. As a subject-like structure, the concept of science refers to a knowledge entity or a system of cognition which, from being an opinion, has become an *episteme*. Scientificity, here, does not denote one or another domain of knowledge or expertise but a degree (the highest degree) of cognition that every domain of knowledge seeks to attain. For Hegel, science is the totality of its components. Developments in the body of knowledge, for him, constitute stages in the development of the Spirit. Rather than being judged as true or false, they must be considered in terms of more or less mature, with each given developmental stage including those that came before it.

So when a knowledge entity evolves into its realization as science, this does not take the shape of linear progress, but instead of dialectical movement, that is to say, a cyclic development of a subject-like structure, which negates its own point of departure and returns to it on a different level, through a process of mutual negations. This yields a stable system that, staying in constant motion, avoids fixation (Yovel, 2001).

Hegel's method, to conclude, describes the developmental totality of a system that retains all the fundamental achievements made in the process. The realization of absolute knowledge approaches itself through opposites and by means of negation: through plurality and otherness. Hence, this development requires an initial resistance of a body of knowledge to otherness, followed, later on, by recognizing other entities of knowledge that are relevant to itself and thereby recognizing itself for the sake of its ongoing development.

Hegel's dialectic is a general theory of scientific knowledge. It corresponds to many aspects of the post-positivist philosophy of science. According to Thagard (1982): “each stage of the dialectic bears the same sort of complex relation to the previous stage as a scientific theory does to its predecessor” (p. 397). According to Hegel the self-development of the subject (or subject-like structures in this case) is dependent upon recognition by other subjects. Understanding the dynamics of Hegel's dialectical method may lead to a new understanding of the historical development of psychoanalysis, as interdisciplinary in essence, in the process of becoming. Below we will employ the above principles of Hegel's method to discuss how bodies of knowledge outside the psychoanalysis domain affect the latter's dialectical development as a distinct yet simultaneously interdisciplinary domain, a mode of development that is vital and indispensable to it.

## The Dialectical Nature of the Interdisciplinary Encounter Between Psychoanalysis and Non-Psychoanalytic Bodies of Knowledge

Here we illustrate the dialectical development of psychoanalysis in its encounter with non-psychoanalytic bodies of knowledge by looking at three important developments in psychoanalysis: Kohut's self psychology; short-term dynamic psychotherapy, and mentalization-based psychotherapy.

### Kohut's Self Psychology

Kohut started to develop self psychology in the 1960's because of difficulties he and most other therapists were having in treating certain patients with so-called narcissistic disorders: issues concerning self-esteem, self-equilibrium, self-regulation, and patients' very core sense of being. Analysts usually addressed these demanding, frustrating, and frequently grandiose clients by interpreting their constant demands on the analyst as stemming from defenses against unconscious aggressive and sexual Oedipal feelings directed toward the analyst. These interpretations usually enraged or depressed these patients, leading analysts, beginning with Freud, to conclude that they could not be analyzed (Tobin, 1991).

Kohut's self psychology (Kohut, 1971, 1977, 1984) foregrounded the power of empathy. He believed that any attempt to understand a patient must have its beginnings in empathy, and he called for the existing psychoanalytic practice to incorporate this insight. In his first major published article, Kohut challenged traditional psychoanalytic practitioners. He announced that the psychoanalyst's job should consist of more than the passive contemplation of the patient's free associations and the subsequent analysis of their resistance. Kohut believed that only by imagining ourselves in the patient's place employing *vicarious introspection* can we bring to life unknown inner experiences. Unlike the mainstream psychoanalysis of his time, which focused on the transference, the unconscious, and recollection, Kohut proposed a method of *empathic validation*, enabling the therapist to validate the patient's description. Kohut rejected interpretation as psychoanalysis' exclusive tool and employed extended empathic interventions to confirm the patient's perception. It is the therapist's task, he believed, to give the patient a sense of the therapist's identification with her or his feelings and to show them their understanding. Any other psychotherapy is at risk of making the patient feel ununderstood, dealing a serious blow to their narcissism.

However, the notion of empathy had already come to be seen as part of Carl Rogers' humanistic method and considered a foreign element by psychoanalysis. Rogers (1942) had been treating patients by using empathy in the 1940's. Kohut and Rogers worked at Chicago University, and although the two never met, they knew about each other (Kahn and Rachman, 2000). The problem, however, was that Rogers had developed his therapy as an alternative to psychoanalysis. On its face, the two methods seemed to clash since psychoanalysis posited that the most important therapeutic process was uncovering the unconscious and insight based on interpretation, not on empathic validation.

Indeed, Kohut was critical of non-analytic and non-interpretive psychotherapeutic counseling, such as the humanistic approach. He likened such psychotherapeutic methods to the work of a repairman who manages to get his old alarm clock to work. Knowing nothing about clocks, all he actually did was to clean it up and oil the internal mechanism (Kohut, 1978, p. 525).

Kohut faced a problem: Could he identify psychoanalysis with an approach which he rejected and criticized? His ideas, indeed, met with strong opposition to begin with. They were taken to clash with psychoanalysis' most fundamental assumptions. Kohut was accused of mocking Freud's core values, appropriating concepts, populism, superficiality, subjectivity, ignoring the unconscious's role, rejecting the scientific method, and turning psychoanalysis into a one-dimensional method (Brenner, 1968; Stein, 1979; Moses, 1988). And yet, part of the psychoanalytic community welcomed his ideas warmly (Menaker, 1978; Schwartz, 1978). As time went by, even the most conservative institutions came to include them in their training programs. Kohut's body of work has proven to have a tremendous impact on the clinical theory and practice of psychoanalysts over the last decades (Carr and Cortina, 2011).

We would like to argue that Kohut's eventual embrace by the psychoanalytic establishment resulted from a dialectical maneuver of self psychology. Empathy was not really foreign to psychoanalysis: Freud referred to it several times in his texts on the joke (Freud, 1905) and group psychology (Freud, 1921). That said, he never used empathy as a significant analytic tool. This may have been due to Freud's desire to cast psychoanalysis as a scientific body of knowledge. Rather than introducing Rogers' “alien” element of empathy, which the psychoanalytic community rejected, Kohut showed that the scientific tradition of psychoanalysis itself implied it. He believed that psychoanalysis could not do without empathy and that it, moreover, was already active in psychoanalytic practice. He showed that empathy cohered with psychoanalysis in its basic function of data collection for the improved understanding of the patient's unconscious dynamic (Kohut, 1959). Kohut (1975) argued that empathy, being a tool for data collection just like the microscope assists the physician to examine a patient's blood, confirmed the scientific nature of psychoanalysis. Meanwhile, to ensure the status of psychoanalysis' distinct, autonomous identity, he also criticized non-analytic and non-interpretive forms of psychological counseling like humanistic psychotherapy (Kahn and Rachman, 2000).

We can then conclude that the component of subjective empathy (self), which was rejected and alienated (other), returned (self) following the negation of its negation, but – through sublation – rather than coming back in the very same form, it returned not as an emotive function but in a new, sublated form. In this process, the idea of empathy became part of psychoanalytic tradition, its definition of the unconscious, and scientificity. Kohut's simultaneous rejection of non-analytic approaches made self- psychology's entrance into mainstream psychoanalysis possible. The distinction between them and us had been preserved, even though*, and because*, this development had been prompted by an encounter with an ostensibly foreign element. This tension between autonomy and dependence was vital in the emergence of psychoanalysis' dialectic identity and its development as a body of knowledge.

### Short-Term Dynamic Psychotherapy

At the same time as Kohut was attempting to change the face of psychoanalysis by introducing the concept of empathy, another no less daring attempt to change the psychoanalytic landscape was underway: the introduction of short-term dynamic psychotherapy (STDP).

The spread in the course of the 1970's and 1980's, and even before, of competing forms of non- analytic short intervention such as planned short-term therapy [an example of such non-analytic new psychotherapies are Milton Erickson brief and strategic model of psychotherapy (Erickson, 1954) and the Strategic family therapy by Haley (1963)], which offered a written protocol and highly technical approach designed for brief treatments, was quite remarkable. Many of the new methods registered an achievement that gave them a distinct edge over psychoanalysis: Not only were they quicker and cheaper, but they were also supported by research that proved their efficacy (Lemma et al., 2010).

The originators of STDP looked beyond the psychoanalytic world and were willing to respond to these presented challenges. In the main, short term therapy was developed during the 1980's and 1990's by several key psychoanalysts: Mann in Boston (Mann, 1973; Mann and Goldman, 1982), Malan in London (Malan, 1963, 1976), Sifneos in Boston (Sifneos, 1972, 1979), and Davanloo in Montreal (Davanloo, 1978, 1980). Additional founders include Donovan (1987), Gustafson (1984), Strupp and Binder (1984), and Luborsky (1984). These orthodox psychoanalysts were looking for solutions to needs that the classical approach failed to meet and sought to cope with its limitations (Mann and Goldman, 1982).

Short term therapies seemed to breach all-important psychoanalytic assumptions at once: free associations were replaced by focused therapy; neutrality and evenly-suspended attention - two fundamental analytical attitudes recommended by Freud (1912, 1915)–were substituted by therapists' active and directed interventions; the structural change was replaced by resolving a central conflict.

These changes raise the question of what elements of classical psychoanalysis we deem to be essential. How were psychoanalysts persuaded to, say, try and resolve an Oedipus complex within the span of fifteen meetings? How did they abandon free association for the sake of focused therapy? What made them become more directly involved, setting aside the pivotal psychoanalytic mode of therapeutic neutrality? These questions can be explained by means of Hegelian dialectics.

First, supporters of the new method pointed out the roots of the technique within the psychoanalytical tradition, particularly in its founder's works. Freud's early treatments were very short, compared not only to today's psychoanalysis but even in terms of today's dynamic short-term analysis. Freud met with Katarina (Freud and Breuer, 1955/1895) only once and yet regarded the meeting as a psychoanalytic session. Gustav Mahler's consultation with Freud was also limited to one session (Jones, 1955; Reik, 1960).

Second, supporters of the approach argued that there were no clashes between a time-bound therapeutic setting, on the one hand, and the psychoanalytic approach to the psyche, its theoretical model, and associated therapeutic techniques. They emphasized that STDP was more effective in achieving analytic objectives than long-term treatment. That is to say, using STDP is not merely a compromise imposed by necessity but rather a better implementation of classical treatment. Moreover, proponents of STDP strictly stuck to employing psychoanalytic terminology and jargon.

One of the most representative examples of STDP is Mann (1973) “Time-Limited Psychotherapy.” Mann limited therapy to a series of twelve sessions following an initial assessment. The idea behind this was to turn time into an active psychoanalytic-therapeutic component. Termination, and hence the time limit, can serve as the main lever for the intensive, fast mobilization of processing and change. The time limit introduces the reality principle into the therapeutic space as opposed to the pleasure principle. This rallies the forces of the ego. Mann also argued that the therapeutic focus on a “main issue” (a problem the patient has been experiencing early on in their life), was a working principle that cohered with classical psychoanalysis. Mann did not seek to replace psychoanalysis; he intended to refine and develop it.

We witness how short-term dynamic psychotherapy restored elements to psychoanalysis that were initially negated. When the negation of elements like therapeutic focus, active position, and especially short term, is negated, they do not return to their previous identity; through sublation, they become more complex and lead to theoretical development. The method becomes relevant and accessible to a larger population, including public mental health clinics that cannot offer long-term therapies. This would not have happened without dialectic development. Here the tension of the interdisciplinary encounter with the therapeutic other and the psychoanalytic tradition's organic growth allows for the mainstream to accept the new development.

### Mentalization-Based Treatment (MBT)

Another change in psychoanalysis occurred in the 1990's. Confronting intensified psychological problems and an access of personality disorders, psychoanalytic communities were prompted to introduce unprecedented change in their modes of treatment while also remaining loyal to the approach from which they had developed.

Mentalization-Based Treatment emerged especially in the context of a growing need to address borderline disorder (according to the DSM definition), given classical dynamic-psychoanalytic approaches' unsatisfactory response. Over time it became clear that impairment of the ability to mentalize entails various emotional disturbances and other symptoms, and this method became widely used.

MBT was developed by British psychoanalysts Peter Fonagy, Anthony Bateman, and Mary Target (Bateman and Fonagy, 2004a,b; Bateman and Fonagy, 2006). It is an approach that links classical (Winnicott) and contemporary psychoanalytic (relational) theories, on the one hand, with research approaches in the field of developmental (Bowlby's attachment theory) and cognitive theory (Baron-Cohen's theory of mind), on the other. It is based on the cognitive-developmental theory of mind and assumes that we intuitively create preconceptions and explanations of behavior from infancy. This mentalization includes an ability to think about thoughts, beliefs, emotions, and wishes – our own and those of others, and understand that these internal events variously affect our own, as well as others' behavior. A rigid and non-mentalizing position will be reflected in monolithic, one-track thinking, while a mentalizing attitude is manifested in the ability to raise several alternative possibilities (Diamant, 2008).

The approach also refers to Winnicott when it argues that mentalizing ability depends on how the caregiver reflects the infant's experience to it. The caregiver shows the infant that what the latter sees reflects its own feelings rather than those of the career herself. This reflection allows the infant to understand that what it feels is not the same as others.

Another point of reference for the theory of mind is Bowlby's developmental theory. It claims that the most significant factor in the development of mentalization is secure attachment relations. Having experienced a secure basis, the infant can gain confidence to explore the world – not only around it but also the inner world, of self and others alike.

Though this approach is considered to be psychoanalytic, it implements interventions that are more reminiscent of cognitive-behavioral methods. Fonagy and Bateman argue that in the case of borderline personality disorder, therapy must focus on enhancing the ability to mentalize rather than encouraging insight through interpretations of the transference. To achieve healthy mentalization, the therapist enables the patient to understand things differently and from a number of viewpoints. Being given impulsive reactions to their emotions, borderline patients are asked to suspend their reactions and thoughts. They are required to process their emotions more appropriately and cultivate a better understanding of other people's perspectives.

So classical psychoanalytic practices are significantly different from MBT. In this case, too, the dialectical process can explain a great deal about how they have come to interrelate, with MBT continuing to identify itself as a psychoanalytic approach, acknowledging its roots in the tradition (especially in object relations theory). Fonagy and Bateman in fact, claim that every therapeutic act includes elements of mentalization so that MBT is not all that innovative. Psychoanalysis, they argue, has always been about recovering the ability to mentalize. It allows the patient to think or reflect on their actions and take an interest in and observe their own and others' consciousness in the secure attachment of the therapeutic relationship. According to Allen (2006), mentalizing is developing awareness of the connections between triggering events in current attachment relationships and previous traumatic experiences. Also cultivating awareness of the impact of one's behavior on attachment figures, an idea originated in Freud's ideas: “Remembering, repeating, and working-through” (Freud, 1914–1958). Allen also indicates that the concept of psychological mindedness is linked to that of insight. Mentalizing highlights the process by focusing on mental activity while insight emphasis the content.

Through its emphasis on cognition and thought processes and thought about a thought formerly seen as extraneous to psychoanalysis, the current presence of cognition in the psychoanalytic mainstream no longer takes its cognitive-behavioral form or its older roots. Now cognition flourishes as an integral component of psychoanalysis, fitting in with its developmental theory, its psychopathology, and therapeutic practice. Thus, for instance, we can see how transference relations have been transforming a classic response to the interpretation of unconscious conflict: a systematic effort is now underway to develop the patient's ability to look at her or himself and others and to build an intelligent look at interpersonal experiential contexts that will help them regulate themselves emotionally. Unlike classical psychoanalysis, this approach abandons the relatively avoidant therapeutic position and the use of in-depth interpretations that involve historical aspects. This model, instead, assumes a more structured and active therapeutic position. It ignores unconscious contents for the benefit of conscious or near-conscious ones. Focusing exclusively on the patient's present mental condition (thoughts, emotions, wishes, desires), the therapist aims to establish the foundations of mental states.

We can put this as follows in Hegelian dialectical terms: While development grounds itself as an outcome of the classical tradition (by viewing pathology as an injury sustained in early attachment relations and in internalized object relations – in theory – and the transference, in practice), the big changes the tradition has undergone are conspicuous: self parts originally negated as being foreign – mainly cognition (in addition to focusing on the present, and therapists' active intervention, in clinical practice) – have been received back in non-identical form (i.e., mentalization). Even though in this move, the cognitive component of psychoanalysis has been restored through sublation, linking between cognition and behavior – a development ostensibly directly deriving from cognitive-behavioral approaches - the mainstream psychoanalytic approach explicitly rejects behaviorist interventions and safeguards its own focus on the patient's consciousness rather than their behavior (in other words, the intra-psychic aspect). Specific components have to keep being rejected as extraneous to enable distinct, though not fully independent, identity formation. This preserves a dialectic tension in the identity of the approach and its body of knowledge.

To conclude: investigation and observation, in a dialectic development, starts off at a certain point; this process of self-examination exposes inner contradiction, and this contradiction leads to another, new position. This new position negates the previous one, issues from it, and advances from it. Thus, in this process of sublation, the earlier condition, its negation, as well as the new condition are all included. In this dialectic, the two sides of the dialectical tension do not merely coexist, they actually entail one another: necessarily and methodically. That is to say: one does not exist and has no value in the absence of the other (Shalgi, 2009).

It could be said that when assimilating alien concepts or constructs and attributing a new status to them, the risk is to multiply theoretical constructs which refer to the same piece of reality or phenomenon. Katzko (2002) calls this shortcoming “The Uniqueness Assumption” (263):

“The uniqueness assumption typologies an observational level of discourse to reflect theoretical distinctions… Another experimenter, manipulating a different set of variables and using the uniqueness assumption to explain the data will by definition create a theory different from the first. The seed is now sown for a proliferation of mutually exclusive theoretical terminologies” (p. 265).

This is all truer to grand theories in psychotherapies who rarely use objective, independent evidence. Instead, data are part of the theory and not different from the theory. Theories that endorse previously negated elements implicitly support the uniqueness assumption by not addressing other possible influences if they are not part of their theoretical model. We cannot fully address this problem here, but we would like to mention that Katzko expects psychological research to follow the rules of strict science. This expectation is, of course, highly controversial in our field and a topic of endless debates. Besides having an empirical scientific side, psychology theory (especially psychotherapy) also offers a net of meanings, an ethic, a way to live in the world (Makari, 2000). This will seem from a scientific perspective to be unsystematic but take meaning through historical analysis.

## The Scientific Status of Psychoanalysis and Shifts in Epistemological Positioning

One of the main controversies within psychoanalysis is its scientific status. Here we would like to address the relationship between this controversy and dialectics briefly.

We have described the dialectics between psychoanalysis and non-analytic theories, but there is also another important dialectic in the field of the epistemology of psychoanalysis. The controversy over the scientific status of psychoanalysis cannot be fully addressed here, but it is important to note some important shifts in epistemological positioning that have occurred and the relation of this controversy to dialectics.

Hegel's dialectics epistemology is not just a theory of general knowledge but also a theory of scientific knowledge. As mentioned before, for Hegel a knowledge entity develops as a subject-like structure and comes into being in the same manner as self-consciousness. For Hegel, science is the totality of its components. Developments in science constitute the developmental stages, each one including those that came before it.

Up until the 7o's, most psychoanalysts followed Freud's scientific worldview and were committed to the idea that psychoanalysis is a science, and that meant devising a mechanistic theory to explain normal and abnormal thought (Basch, 1993). In the last decades, there was a radical shift, and many scholars suggested modern Hermeneutics and post-modernism as better epistemologies. Others seek a more intermediate position between science and hermeneutics (Makari, 2000; Negri et al., 2019). Fusella (2014) argues that psychoanalysis has situated itself among the other disciplines as a hybrid science, not quite a pure hermeneutic on the one hand and not quite a pure science.

The change in epistemological positioning in the scientific discourse can also be seen in the disagreements about empirical evidence and the evaluation of psychoanalysis [e.g., Hoffman (2009) and Safran (2012), response]. Hoffman thinks that systematic empirical research on psychotherapy process and outcome is less relevant for psychoanalysis than the traditional case studies. In contrast, Safran (2012) describes a middle ground approach to science which recognizes that “science has an irreducibly social, hermeneutic, and political character, and that data are only one element in an ongoing conversation between members of a scientific community” (p. 710). While positivism argues for only one path to truth, hermeneutics believes that there is more than one truth. Its interest is in emergent processes and moral commitments to self-reflection and critical thought (Cushman, 2013).

It seems that relational theory has shifted away from realist aspirations or impersonal objectivity to the creative power of human imagination as regards to subjectivity, intersubjectivity, and truth (Elliott and Spezzano, 1996). Moreover, some post-modern discourses have sought to attack the scientific worldview and undermine scientific truths in order to undermine science (Kuntz, 2012). However, it is to the credit of the post-modern thinkers in relational psychoanalysis that they have insisted on rejecting radical anti-scientist post-modernism as an appropriate epistemology for psychoanalysis. In a manner often seen in dialectical development, the relational tradition has, in fact, had a positive impact on certain aspects of psychoanalysis. Post-modern approaches such as the relational approach do not necessarily lead to rejecting data, which are still a central source of intersubjective knowledge. Instead, the very acknowledgment that data necessarily requires interpretation entails a renewed centrality of data themselves. Also, post-modern theories do not necessarily mean that realism is false; the acknowledgment that objectivity is always intersubjectivity doesn't necessarily clash with a contemporary view of science. Osbeck (2019), for example, offers a holistic picture of the scientific project that acknowledges the role of imagination, perspective-taking, and values alongside observation and reason. For her, foregrounding 'the personal' also emphasizes continuity across arts and sciences, the interfaces of which contain the full range of resources for innovative thinking.

We believe that such post-modern approaches are dialectical by their nature in incorporating antagonists and contradictions. Here too, dialectics appears to be relevant. For example, classical psychoanalysts who tend to perceive psychoanalysis as a scientific discipline and to endorse realism and the correspondence theory of truth vehemently oppose scientific evidence of all sorts, particularly when it derives from non-analytic theories [as in Blass and Carmeli (2007) criticism of neuropsychoanalysis, and Green (2000) criticism against infant research]. At the same time, the relational theoreticians were the first to endorse psychoanalytically informed infant research based on systematic observations on parent-infant interaction outside psychoanalysis. Benjamin (2013) writes:

“…. infancy research electrified me. What awesome possibilities it seemed to open up, is what I felt when I first encountered the work of Stern (1974a,b) and Beebe (Beebe and Stern, 1977) in 1978. Face-to-face play was the primary illustration of how mutual recognition is possible so early! …As it now seemed that all roads were leading to recognition and intersubjectivity, somehow I had to get them all on the same map” (p. 4).

Here again, objective science was negated but then return in a different form as representing intersubjectivity, a term that infant researchers like Stern (1974a) use for lack of a better word. Paradoxically, infant research is incorporated in a post-modern approach not only for its systematic and evidence-based methods but for its detailed description of recognition and intersubjectivity. Likewise, classical psychoanalysts show indifference to non-analytic theories but only after the psychoanalytic theories are well-founded. Steiner (2000) notes that “there is no doubt that extra-analytical observations played a certain role in confirming and refining, at times, Freud's and the first child analysts' observations and hypotheses concerning the chronology of the development of the internal life of the baby and the child” (p. 10). Steiner shows that in the phase of imagining a new infant, Winnicott, Klein, Lacan, and others used non-analytic theories to validate their new theories of development.

## Future Application

Mainstream psychoanalysis has, as said, invested significant resources to defend its boundaries from external influences. This jealous self-protection requires an investment in keeping the self and others apart. Any attempt at development within the discipline requires proof that new ideas don't smuggle in foreign elements – which will meet rejection. However, we have seen that from a dialectical perspective, any development, any movement ahead, necessarily involves such foreign elements. This is a foreignness that should not be considered only extraneous to psychoanalysis.

In this paper, we offer a new perspective on interdisciplinarity in the psychoanalytic clinic. Rather than either isolationism or an externally imposed alienated unity in the face of the other, we have sought to reflect on psychoanalysis' encounter with other theories in terms of a dialectical movement within psychoanalysis itself. Here the other, non-analytical approach is seen to constitute an integral moment in the development of psychoanalytic knowledge.

Distinction and emphasizing salient differences between the schools in the field of psychotherapy is made in purpose to sustain professional-cultural identity. Psychoanalysis needs to hold convictions about what it believes and what it rejects for a stable and robust identity. As a result, mental barriers are formed which keep out the threat and keep the subject at a safe intellectual distance. This phenomenon is not unique to psychoanalysis; Wachtel (2010, 2018) sees the field of psychotherapy as divided between “tribal organizations” entangled in a culture war, something more like an ethnic conflict with its attendant us-vs.-them feelings than an intellectual or scientific discussion. Differences are polarized; caricature and stereotype abound; each side is intensely attached to its own way, and self-definition is achieved by diminishing the other. “What lies outside might be not only not noticed but actively rejected since it is associated with a point of view that is derided and disdained as ‘other,'” in Wachtel's words (Wachtel, 2010, p. 407). it seems that no school of therapy appears to have a monopoly on dogmatism or therapeutic insensitivity (Shedler, 2010).

Authors writing about fragmentation, disunity, and the crisis of the field, have been facing the problem from perspectives ranging from political viewpoints to rhetorical, via theoretical-methodological, historical, educational, and meta-theoretical levels of inquiry and also as a sociocultural phenomenon including organizational processes and traditional communities (Gaj, 2016). Many clinicians have sought to offer a solution to this disunity in the field; at the theoretical level by trans-theoretical approaches (Prochaska and DiClemente, 2019) and at the research level of evaluation findings by developing near-optimal systematic statistical prediction rules that should be used in preference to intuition (Dawes, 2005). Hegel's dialectics may offer another solution that does not seek unity in conformity of method or theory.

We believe that actively encouraging the position of reflection and self-skepticism and openness to and acknowledging the other, by knowing the dialectic, is of great importance: “Through mutual recognition, each discipline moves closer to appreciating the value of the other, and this process is what advances knowledge. Like spirit, which seeks recognition from the other so that it may recover its lost alienated desire, mutual recognition provides mutual validation and acceptance, which opens up further communication and dialogue” (Mills, 2012, p. 192).

Hegel's dialectical development of the subject or of Subject-Like Structures like psychoanalysis is a description of how things evolve naturally as we constitute ourselves and our knowledge. It is true that according to the dialectics, only in the affirmation of identity, difference, and then subsequently in sublation can the process of knowledge proceed. At the same time, developments in psychotherapy in general and in psychoanalysis, in particular, occur in different ways through exposure to the other (for example, Peri Herzovich and Govrin, 2021). According to this, we believe that when psychoanalysis can identify itself by finding itself in its other and by finding the other within itself – which is tantamount to acknowledging its own self-difference or alienation – it will have an ability to expose itself to other theories to conduct a respectful and stimulating interaction. This would in fact, be its way of maintaining its separate identity, exactly through acknowledging extra-analytical bodies of knowledge and its own position vis a vis them.

Psychoanalysis will reap multiple benefits from its interest in these different theories. It will get to know itself better, allow for self-criticism, questions, doubt, and it will be open to consider its own shortcomings. This will help it avoid paralysis, dwindling creativity, and growing irrelevant and outdated.

In an era marked by frequent change, psychoanalysis cannot afford to remain stuck. To stay in contact with developments and remain relevant, it needs to foster its ability to find sustenance outside itself – as long as that sustenance does not threaten its continued existence. We suggest that rather than directing its efforts to put up walls and ignoring other, non-psychoanalytic approaches, psychoanalysis should look for its commonalities with them – first, as it looks inward and then to confidently open itself to such encounters and even encourage them. According to the dialectical process, growth is enabled by the other but confirms the self in so far as it must be able to recognize the other.

To conclude, while psychoanalysis defines itself by reference to what it is not, its development necessitates an ability to recognize that what is extraneous to itself is also part of itself. When it acknowledges its other, psychoanalysis recognizes its own otherness or its multiple nature. This forms simultaneously, and dialectically, a recognition of its own unity.

## Data Availability Statement

The original contributions presented in the study are included in the article/supplementary material, further inquiries can be directed to the corresponding authors.

## Author Contributions

The article forms part of a doctoral study at Bar Ilan University in the Program for Hermeneutics and Cultural Studies undertaken by YP. AG was the supervisor of this doctoral study. Both authors contributed to the article and approved the submitted version.

## Conflict of Interest

The authors declare that the research was conducted in the absence of any commercial or financial relationships that could be construed as a potential conflict of interest.
